# Influence of Piracetam on Gliclazide—Glycated Human Serum Albumin Interaction. A Spectrofluorometric Study

**DOI:** 10.3390/molecules24010111

**Published:** 2018-12-29

**Authors:** Agnieszka Szkudlarek, Jadwiga Pożycka, Małgorzata Maciążek-Jurczyk

**Affiliations:** Department of Physical Pharmacy, Medical University; School of Pharmacy with the Division of Laboratory Medicine, 4, 41-200 Sosnowiec, Poland; jpozycka@sum.edu.pl (J.P.); mmaciazek@sum.edu.pl (M.M.-J.)

**Keywords:** HSA, glycation, AGEs, piracetam, gliclazide

## Abstract

Advanced Glycation End-Products (AGEs) are created in the last step of protein glycation and can be a factor in aging and in the development or worsening of many degenerative diseases (diabetes, chronic kidney disease, atherosclerosis, Alzheimer’s disease, etc.). Albumin is the most susceptible to glycation plasma protein. Modified albumin by AGEs may be more resistant to enzymatic degradation, which further increases the local accumulation of AGEs in tissues. The aim of the present study was to analyze in vitro glycation of serum albumin in the presence of piracetam (PIR) and the gliclazide (GLZ)-glycated albumin interaction. The analysis of PIR as an inhibitor and GLZ interaction with nonglycated human albumin (HSA) and glycated by fructose human albumin (gHSA_FRC_), in the absence and presence of piracetam (gHSA_FRC_-PIR), was performed by fluorescence quenching of macromolecules. On the basis of obtained data we concluded that under the influence of glycation, association constant ($K_{a}$) of gliclazide to human serum albumin decreases and GLZ binds to HSA with less strength than under physiological conditions. PIR strongly inhibited the formation of AGEs in the system where the efficiency of HSA glycation was the largest. The analysis of piracetam influence on the GLZ-glycated albumin interaction has shown that piracetam increases the binding strength of GLZ to glycated albumin and weakens its therapeutic effect. Based on the obtained data we concluded that monitoring therapy and precautions are required in the treatment when the combinations of gliclazide and piracetam are used at the same time.

## 1. Introduction

Diabetes, as the only noninfectious disease, was recognized by the UNO as an epidemic of the XXI century. The International Diabetes Federation (IDF) estimates that the number of people with diabetes in 2035 increases to 592 million [1]. Due to the long-term course of the disease, damage, dysfunctions, and insufficiency of many tissues and organs occur. The main factor responsible for the organ complications of diabetes, i.e., macro- and microangiopathy, is an increase in the concentration of reducing sugars in the blood. The carbonyl group of reducing sugar (including glucose, fructose, and galactose), in a series of transformations called Maillard reactions, reacts with the primary free amino group of the NH_2_-terminal residue of the protein or the lysine (Lys) and arginine (Arg) residues, causing their glycation [2,3]. The protein glycation process is multistep; in the last stage, Advanced Glycation End-Products (AGEs) are created. The formation of AGEs accelerates oxidative stress [4]. Due to the presence of AGEs in diabetic patients’ blood, in the late complications of diabetes (retinopathy, nephropathy, neuropathy, and coronary atherosclerosis) not only hyperglycemia, but also glycosylated connections take place. This fact has been confirmed by the scientific studies on the animal models and in vitro glycated albumin coming from human or bovine [5,6,7,8]. AGEs can modulate the signaling pathways in the cell and influence the inflammation mediators involved in the pathomechanism of diabetic complications [9]. Albumin is the most susceptible to glycation plasma protein, where its concentration reaches 526–725 μmol/L (35–50 g/L) [10]. It is a highly hydrophilic, globular simple protein having a negative electrical charge at physiological pH. As a proprotein, it is synthesized by liver cells in an amount of 10–15 g per day, which is −10% of the total protein synthesis in this organ [11]. The final form of albumin is formed by an enzymatic process with the cleavage of the hexapeptide from the *N*-terminus of proalbumin [12]. The human serum albumin (HSA) molecule consists of 585 amino acid residues that form a pleated 3-chain polypeptide chain with 67% of residues forming α-helix structure, 23% are stretched polypeptide fragments, and 10% are β-turns [11,13]. The amino acid sequence contains 17 disulfide bridges and one free sulfhydryl group -SH (Cys-34). HSA contains in its amino acid composition a single tryptophanyl residue at position 214 (Trp-214) and 17 tyrosyl residues (Tyr-30, -84, -138, -140, -148, -150, -161, -263, -319, -332, -334, -341, -353, -370, -401, -411, and -497) [14], which is very important when using test methods with fluorescent probes.

**Figure 1:**
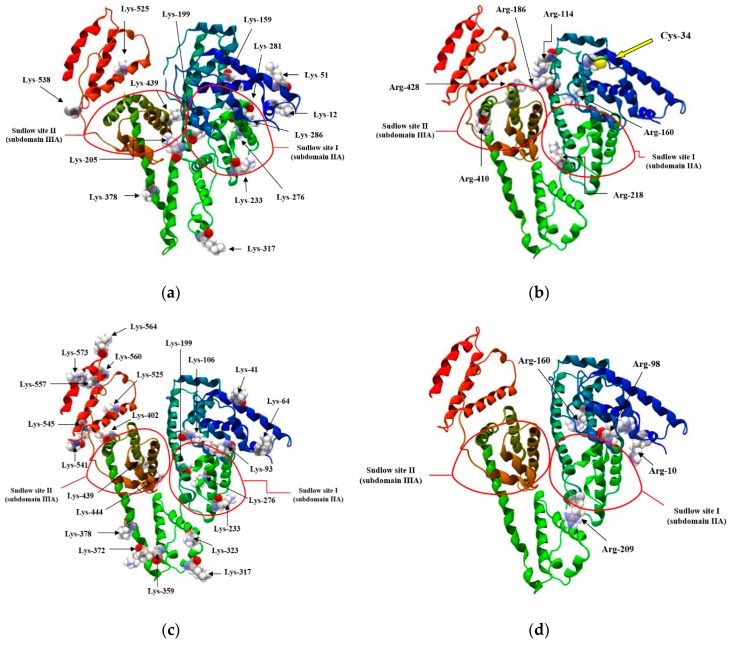
Human serum albumin drug binding sites (i.e., Sudlow’s site I and II) with the location of main (**a**,**c**) lysine (Lys) and (**b**,**d**) arginine (Arg) residues involved in (**a**,**b**) in vivo [16] and (**c**,**d**) in vitro [17] glycation.

Lysine (Lys) and arginine (Arg) residues and a free thiol group of cysteine (Cys-34) are the most sensitive amino acid residues for in vivo and in vitro HSA glycation due to their strong nucleophilic character [3]. Figure 1 shows the specific binding sites for exogenous and endogenous substances in the structure of HSA (drug binding sites) (i.e., Sudlow’s site I and II) [15]) with the location of the main glycation sites involved in in vivo (Figure 1a,b [16]) and in vitro (Figure 1c,d [17]) glycation processes.

Taking into account the different mechanisms of AGEs formation, it is not surprising that they are a heterogeneous mixture of compounds with different molecular weights and characteristic properties. AGEs can occur as low molecular weight (LMW-AGEs) and high molecular weight (HMW-AGEs), which were detected in the plasma of people with chronic renal failure [18]. AGEs can also be classified in terms of their ability to show fluorescence and the ability to form cross-links between proteins [19]. The first class of compounds—exhibiting cross-linking, absorption, and fluorescence properties—includes pentosidine (PENT), which is an indicator for the assessment of tissue damage under the influence of glycation [20]. The second class of compounds, the nonfluorescent, cross-linking AGEs, are the main factors leading to the in vivo formation of protein–protein cross-links. Among them, the most well-known group of compounds is the imidazole cross-linking structures, including derivatives of glyoxal-lysine dimer (GOLD) and methylglyoxal-lysine dimer (MOLD). The presence of these compounds was found in the protein of the lens and human serum [20]. The third class are nonfluorescent, non-cross-linking AGEs structures that act as biological receptor ligands, initiating cell signaling and inducing oxidative stress in the tissues. To the following compounds belong to these groups, pyrroline (Pyr), N^Ɛ^-(carboxymethyl)lysine (CML), N^Ɛ^-(karboxyethyl)lysine (CEL) and imidazolone with 3-deoxyglucosone (3-DG), methyloglyoxal (MG), and glyoxal (GL) [21]. In the AGEs, carboxymethyl arginine (CMA), carboxymethyl cysteine (CMC), and carboxyethyl cysteine (CEC) are also included. The presence of CMA was found in collagen, while CMC and CEC were identified in diabetics’ serum protein [22].

The key feature of AGEs is the ability to cross-react with other proteins as a result of disrupting their structure and functions. Proteins modified by AGEs may be more resistant to enzymatic degradation, which further increases the local accumulation of AGEs in tissues [23]. By binding to specific receptors (Receptor for Advanced Glycation End-Products, RAGEs) located on the surface of many cell types (including phagocytes, hepatocytes, endothelial cells and smooth muscle, vascular walls, and nerve system cells), AGEs activate intracellular signaling pathways leading to the creation of, for example, reactive oxygen species [24]. The AGEs–RAGEs interaction has also been shown to activate the nuclear transcription factor κB, which affects the expression of inflammatory response genes (e.g., cytokines) and causes chronic damage of almost every tissue in the body [25].

Due to the suggestion that AGEs take place in etiopathogenesis of many diseases [26,27,28,29], inhibition of the formation of AGEs by the use of substances (AGEs inhibitors) is a promising therapeutic goal. Intensive search for effective and safe drugs that inhibit glycation of proteins is still ongoing. Aminoguanidine as an inhibitor is too toxic for use in patients with diabetes.

**Figure 2:**
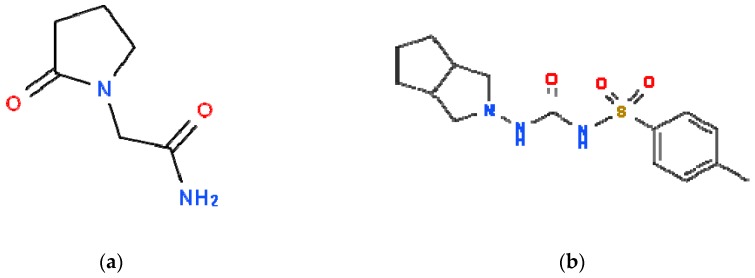
Chemical structure of (**a**) piracetam (PIR) and (**b**) gliclazide (GLZ). The structures were downloaded from http://www.chemspider.com.

Elderly patients undergo multidrug therapy and they may take piracetam (PIR) and gliclazide (GLZ) simultaneously. PIR (Figure 2a) has anticonvulsant and neuroprotective properties and is reported to improve neural plasticity. Its efficacy is documented in cognitive disorders and dementia, vertigo, cortical myoclonus, dyslexia, and sickle cell anemia although the clinical application in these conditions is not yet established [30]. PIR affects the vascular system through the reduction of erythrocyte adhesion to vascular endothelium, hindering vasospasm, and facilitating microcirculation [31]. GLZ (Figure 2b) is an oral antihyperglycemic agent used for the treatment of non-insulin-dependent diabetes mellitus. Based on the pharmacological efficacy, GLZ is considered a second-generation sulfonylurea which presents a higher potency and a shorter half-life [32].

The aim of the present study was to analyze an in vitro glycation of serum albumin in the presence of piracetam (PIR) as a potential glycation inhibitor and its effect on gliclazide (GLZ) binding to glycated albumin using fluorescence quenching method.

## 2. Results and Discussion

### 2.1. Piracetam as an Inhibitor of Human Serum Albumin In Vitro Glycation

Advanced Glycation End-products (AGEs) are a heterogeneous mixture of compounds with different properties that play a significant role in the pathology of diabetes and its associated diseases. Melanoidins, which belong to AGEs, fluoresce [8]. In order to prove the impact of piracetam (PIR) on the formation of AGEs in glycated human serum albumin by monosaccharides such as glucose (GLC), fructose (FRC), and galactose (GAL), emission fluorescence spectra of AGEs coming from HSA-monosaccharide and HSA-monosaccharide-piracetam (on the day of the preparation) and created in gHSA_monosaccharide_ and gHSA_monosaccharide_-piracetam (after glycation) were recorded at λ_ex_ = 335 nm, λ_ex_ = 370 nm, and λ_ex_ = 485 nm. The observed fluorescence of AGEs was corrected for the Raman effect of the solvent and then the maximum fluorescence wavelength (λ_max_) and the AGEs fluorescence intensity at this wavelength (F_max_) have been registered.

**Figure 3:**
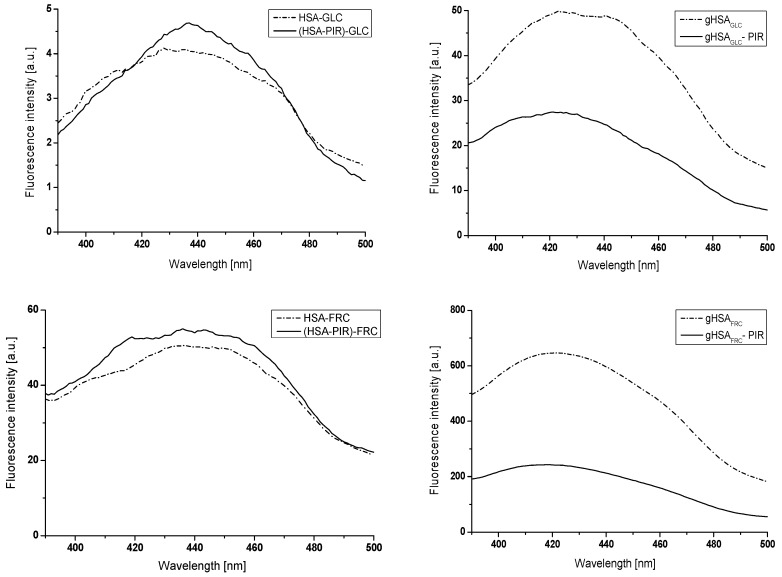

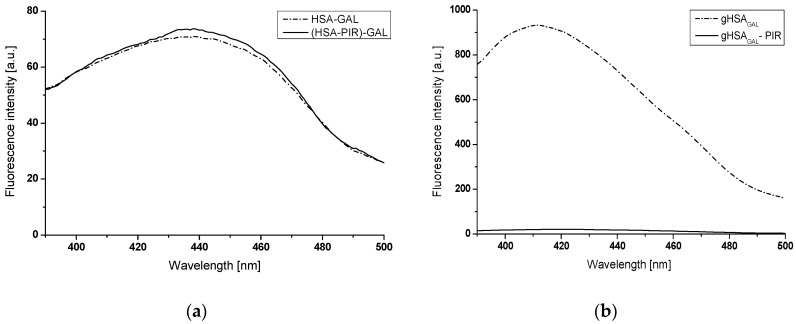
Advanced glycation end-products (AGEs) emission fluorescence spectra registered at λ_ex_ = 335 nm wavelength, (**a**) in a day of solutions preparation (HSA-GLC, (HSA-PIR)-GLC, HSA-FRC, (HSA-PIR)-FRC, HSA-GAL, and (HSA-PIR)-GAL) and (**b**) after 21 days of incubation at 37 °C (gHSA_GLC_, gHSA_GLC_-PIR, gHSA_FRC_, gHSA_FRC_-PIR, gHSA_GAL_, and gHSA_GAL_-PIR), [HSA] 2 × 10^−6^ mol∙L^−1^, [PIR] 5.3 × 10^−6^ mol∙L^−1^, and [GLC, FRC, GAL] 0.05 mol∙L^−1^.

Figure 3 shows the emission fluorescence spectra of HSA glycation products in the presence of glucose (GLC), fructose (FRC), and galactose (GAL), in the absence (HSA-GLC, HSA-FRC, and HSA-GAL systems) and in the presence of piracetam (PIR), at 5.3 × 10^−6^ mol∙L^−1^ concentration ((HSA-PIR)-GLC, (HSA-PIR)-FRC, and (HSA-PIR)-GAL systems), recorded at the excitation wavelength λ_ex_ = 335 nm, in a day of solutions preparation (HSA-GLC, (HSA-PIR)-GLC, HSA-FRC, (HSA-PIR)-FRC, HSA-GAL, and (HSA-PIR)-GAL) (a) and after 21 days of incubation at 37 °C (gHSA_GLC_, gHSA_GLC_-PIR, gHSA_FRC_, gHSA_FRC_-PIR, gHSA_GAL_, and gHSA_GAL_-PIR) (b). The concentration of HSA and monosaccharides were 2 × 10^−6^ mol∙L^−1^ and 0.05 mol∙L^−1^, respectively.

**Figure 4:**
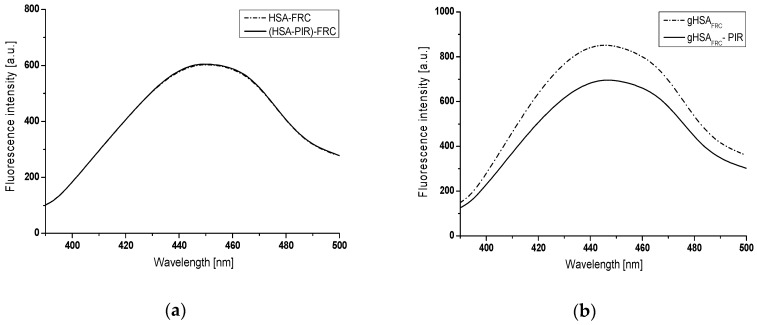
AGEs emission fluorescence spectra registered at λ_ex_ = 370 nm wavelength, (**a**) in a day of solutions preparation (HSA-FRC and (HSA-PIR)-FRC) and (**b**) after 21 days of incubation at 37 °C (gHSA_FRC_ and gHSA_FRC_-PIR), [HSA] 5 × 10^−4^ mol∙L^−1^, [PIR] 1.33 × 10^−3^ mol∙L^−1^, and [FRC] 0.05 mol∙L^−1^.

Figure 4 shows an example emission fluorescence spectra of HSA (physiological concentration 5 × 10^−4^ mol∙L^−1^) glycated products—AGEs in the presence of fructose (0.05 mol∙L^−1^ concentration) with and without of piracetam (1.33 × 10^−3^ mol∙L^−1^), registered in a day of solutions preparation (a) and after 21 days of incubation at 37 °C (b), at excitation wavelength λ_ex_ = 370 nm.

**Figure 5:**
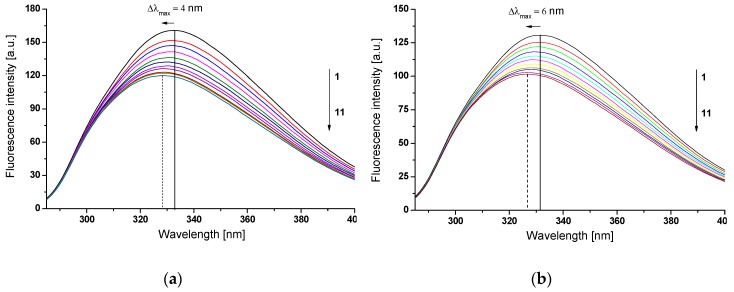
Emission fluorescence spectra of (**a**) HSA and (**b**) gHSA_FRC_ at 2 × 10^−6^ mol∙L^−1^ (1) concentration in the presence of GLZ at 4 × 10^−6^ mol∙L^−1^ (2)–40 × 10^−6^ mol∙L^−1^ (11) concentrations, λ_ex_ = 275 nm; the arrows indicate the shift direction of the maximum fluorescence of HSA and gHSA_FRC_ (Δλ_max_) with the increase of GLZ concentration; t = 37 °C.

Emission fluorescence spectra of glycation end-products (AGEs) recorded in a day of the solutions preparation in the presence or absence of PIR show similar intensity for HSA, both at 2 × 10^−6^ mol∙L^−1^ (Figure 3) and 5 × 10^−4^ mol∙L^−1^ concentration (Figure 4). After 21 days of incubation at 37 °C, an over 11-fold increase in the fluorescence intensity of AGEs in serum albumin in the presence of fructose (gHSA_FRC_) at 2 × 10^−6^ mol∙L^−1^ and 0.05 mol∙L^−1^ concentration, respectively (Figure 3) and 1.4-fold increase in AGEs fluorescence in glycated HSA at physiological concentration (Figure 4) have been registered. On the contrary, only a slight increase in AGEs fluorescence has been observed for HSA (2 × 10^−6^ mol∙L^−1^ and 5 × 10^−4^ mol∙L^−1^) modified by fructose (0.05 mol∙L^−1^) in the presence of piracetam (5.3 × 10^−6^ mol∙L^−1^ and 1.33 × 10^−3^ mol∙L^−1^) (gHSA_FRC_-PIR) after 21 days of incubation (Figure 3 and Figure 4).

The change in AGEs fluorescence intensity registered after 21 days of HSA incubation in the presence of glucose, fructose, and galactose, with and without of piracetam has been calculated as a quotient of glycation products fluorescence at maximum emission in gHSA_monosaccharide_ and gHSA_monosaccharide_-PIR ($\frac{F_{gHSA_{monosaccharide}}}{F_{gHSA_{monosaccharide}-PIR}}$). The quotient $\frac{F_{gHSA_{monosaccharide}}}{F_{gHSA_{monosaccharide}-PIR}}$ has been determined for λ_ex_ = 335 nm, λ_ex_ = 370 nm, and λ_ex_ = 485 nm excitation wavelengths. Obtained results have been collected in Table 1.

Emission fluorescence spectra of HSA glycation end-products (AGEs) in the presence of GLC (HSA-GLC), both GLC and PIR ((HSA-PIR)-GLC), FRC (HSA-FRC), both FRC and PIR ((HSA-PIR)-FRC), GAL (HSA-GAL), and both GAL and PIR ((HSA-PIR)-GAL), at λ_ex_ = 335 nm, λ_ex_ = 370 nm, and λ_ex_ = 485 nm excitation wavelengths show similar intensities in a day of solutions preparation. After 21 days of incubation, higher AGEs fluorescence intensity in HSA glycated by glucose, fructose and galactose compared with AGEs fluorescence intensity from glycated HSA in the presence of piracetam has been registered. This phenomenon may indicate that piracetam has the ability to inhibit the formation of AGEs. The most significant differences were recorded for albumin glycated by galactose, in the absence (gHSA_GAL_) and in the presence of piracetam (gHSA_GAL_-PIR), as indicated on the basis of AGEs fluorescence intensity quotients (Table 1). However, the lowest value of the quotient was obtained for glucose glycated albumin, in the presence and in the absence of piracetam. Based on the in vitro data, it can be concluded that piracetam most strongly inhibited the formation of AGEs in the system where the efficiency of HSA glycation was the largest. Differences in the fluorescence intensity of albumin AGEs are more pronounced for HSA at a concentration of 2 × 10^−6^ mol∙L^−1^ than at a physiological (5 × 10^−4^ mol∙L^−1^) one.

### 2.2. The Interaction of Gliclazide (GLZ) with Nonglycated (HSA), Glycated in the Presence of Fructose (gHSA_FRC_), and Glycated in the Presence of Both Fructose and Piracetam (gHSA_FRC_-PIR) Human Serum Albumin

In order to assess the influence of glycation and piracetam as a glycation inhibitor on the HSA–gliclazide (GLZ) interaction, emission fluorescence spectra of gHSA_FRC_ and (gHSA_FRC_-PIR) at 2 × 10^−6^ mol∙L^−1^ concentration, in the absence (1) and in the presence of GLZ at 4 × 10^−6^ mol∙L^−1^ (2) and 40 × 10^−6^ mol∙L^−1^ (11) concentrations have been registered. Using the same experimental procedures, emission fluorescence spectra of nonglycated HSA in the presence of ligand (control sample) were also registered. Albumin fluorescence has been excited at λ_ex_ = 275 nm and λ_ex_ = 295 nm excitation wavelengths. To identify the shifts (Δλ_max_) of albumin fluorescence maxima in GLZ-HSA, GLZ-gHSA_FRC_, and GLZ-(gHSA_FRC_-PIR) systems, the wavelength corresponding to the maximum albumin fluorescence in the presence of drug at the highest concentration (11) was compared to the maximum albumin fluorescence without ligand (1). Based on the emission fluorescence intensity of HSA, glycated both in the presence and absence of piracetam and nonglycated, the quenching fluorescence curves of gHSA_FRC_, (gHSA_FRC_-PIR) and HSA in the presence of GLZ have been drawn. The course of Stern-Volmer curves (Equation (1), Section 3.6.) allowed us to obtain the information on fluorophores quenching fluorescence by GLZ. From the modified Stern-Volmer dependence (Equation (2), Section 3.6.), the values of $K_{\mathrm{SV}}$ constants in GLZ-(gHSA_FRC_-PIR) system and the fraction of the maximum fluorescence accessible to the quencher ($f_{a}$) have been calculated. Saturation binding curves and binding isotherms were used to determine the nature of the drug’s interaction with glycated and unglycated albumin. From the Scatchard (Equation (3), Section 3.6.) and Klotz (Equation (4), Section 3.6.) curves, the number of drug molecules that bind to one molecule of albumin at equilibrium (n) was also determined. From the Hill’s dependence (Equation (5), Section 3.6.), Hill coefficients have been calculated ($n_{H}$).

#### 2.2.1. The Influence of Glycation on Gliclazide–Serum Albumin Interaction

Figure 5 shows exemplary fluorescence emission spectra of HSA (Figure 5a) and gHSA_FRC_ (Figure 5b) in the presence of GLZ at excitation wavelength λ_ex_ = 275 nm.

Emission fluorescence spectrum of unglycated (Figure 5a) and glycated (Figure 5b) gHSA_FRC_ excited at λ_ex_ = 275 nm and λ_ex_ = 295 nm (data not shown), respectively, has one maximum of fluorescence at λ_em_ = 332 nm (λ_ex_ = 275 nm) and λ_em_ = 338 nm (λ_ex_ = 295 nm). In the presence of gliclazide (GLZ), with the increase of its concentration, the fluorescence intensity of excited fluorophores gradually reduces. It probably means that HSA and gHSA_FRC_ fluorescence is quenched by a gliclazide molecule and the distance between the ligand and protein fluorophore is probably smaller than 10 nm (~80–100 Ǻ) [33] or than 7 nm [34]. This distance makes it possible to emit nonradiative, direct energy transfer to the drug molecule. Using excitation wavelength λ_ex_ = 275 nm, the presence of GLZ caused a blue-shift of both, HSA and gHSA_FRC_ spectra in GLZ-HSA (Figure 5a) and GLZ-gHSA_FRC_ (Figure 5b) systems. The hypsochromic shift of maximum albumin fluorescence (Δλ_max_) caused by the presence of GLZ is associated with a decrease in polarity (increase in hydrophobicity) of unglycated and glycated HSA fluorophores after binding to the ligand. It probably indicates the possibility of hydrophobic interactions between the aromatic rings of the gliclazide molecule and aromatic amino acid rings of the hydrophobic HSA and gHSA_FRC_ cavity within IIA (Trp-214) or/and IB, IIB, and IIIA subdomains, where the tyrosyl residues are also located. A shift (Δλ_max_) of maximum albumin fluorescence caused by GLZ, stronger for gHSA_FRC_ (Δλ_max_ = −6 nm) than for HSA (Δλ_max_ = −4 nm) (Figure 5) have been observed. At the excitation λ_ex_ = 295 nm no shift has been recorded. Similar observations were noticed by Maciążek-Jurczyk et al. [35] during the fluorescence analysis of tamoxifen (TMX) and curcumin complex (CUR) with human serum albumin (HSA). With the increase of ligand concentration, the authors recorded hypsochromic shift of HSA fluorescence band, Δλ_max_ = −18.5 nm and Δλ_max_ = −4.5 nm, in the presence of TMX and CUR, respectively. The change in the fluorescence intensity of HSA and gHSA_FRC_ fluorophores can also be the result of conformational changes in the macromolecule of both, unglycated and glycated albumin due to the binding with GLZ. Monti et al. [36] postulated, based on the circular dichroism data, that the binding of another ketoprofen (KP) molecule to HSA causes structural changes in the albumin macromolecule by disrupting small protein areas already at KP:HSA 1:1 molar ratio. Fluorescence of glycated albumin (gHSA_FRC_) (Figure 5b) in comparison to the unglycated (Figure 5a) showed less intensity (F_max_) and stronger hypsochromic shift (Δλ_max_) in the presence of gliclazide. This phenomenon may indicate presence of some the structural changes of glycated protein that can be manifested by the increase of GLZ binding sites in gHSA_FRC_ hydrophobic properties. Sakurai et al. explained the decrease in the fluorescence intensity of the tryptophanyl residue in glycated HSA (G-HSA) compared with nonmodified protein explained by the transfer of energy from the Trp-214 to the newly formed chromophore in G-HSA [37]. Spectrofluorimetric studies and circular dichroism analysis of secondary and tertiary structure of unmodified and fatty acid free glucose glycosylated albumin suggested that HSA glycation changes the local structure around Trp-214, but does not significantly affect the secondary structure of HSA [38]. The authors observed that the fluorescence intensity of Trp-214 located in glycated albumin in the presence of 6 mol∙L^−1^ guanidine hydrochloride is lower and shifts towards the shortwave direction as compared to the chromophore fluorescence of unglycated HSA in the presence of denaturant. This supports the idea that glycation process influences the tryptophanyl residue environment. Another insight of the authors was the protective effect of glycation on the destabilization of both the secondary and tertiary HSA structure caused by chemical denaturation. Mendez et al. suggested that different fluorescence of the glycated and unglycated tryptophanyl albumin residue may be the result of different degrees of macromolecules hydration.

**Figure 6:**
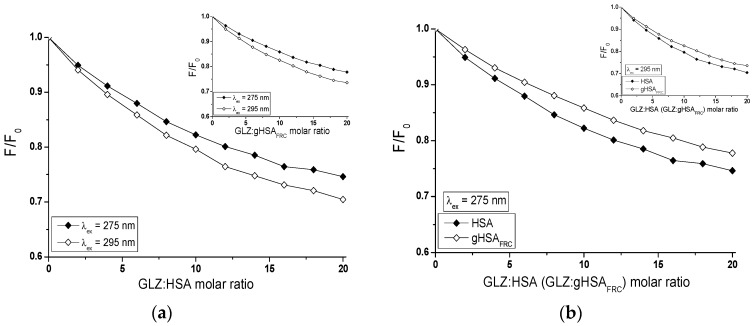
Fluorescence quenching of nonglycated (HSA) and glycated (gHSA_FRC_) human serum albumin complexed with GLZ (4 × 10^−6^ mol∙L^−1^–4 × 10^−5^ mol∙L^−1^). The albumin concentration was 2 × 10^−6^ mol∙L^−1^; (**a**) GLZ-HSA (in the main view) and GLZ-gHSA_FRC_ (in the insert) and (**b**) λ_ex_ = 275 nm (in the main view) and λ_ex_ = 295 nm (in the insert); the error bars are smaller than the symbols.

Fluorescence quenching curves show the dependence of the albumin fluorescence quotient in the presence (F) and in the absence of ligand ($F_{0}$) in function of the molar ratio ligand: HSA. Based on data obtained from the emission fluorescence spectra, the quenching curves of HSA and gHSA_FRC_ (2 × 10^−6^ mol∙L^−1^) fluorescence in the presence of GLZ (4 × 10^−6^ mol∙L^−1^–4 × 10^−5^ mol∙L^−1^) at λ_ex_ = 275 nm and λ_ex_ = 295 nm have been drawn (Figure 6).

The comparison of fluorescence quenching curves of HSA and gHSA_FRC_, in the presence of gliclazide at the excitation wavelength λ_ex_ = 275 nm and λ_ex_ = 295 nm allowed to indicate the fluorophores involved in the interaction with the ligand. Excitation of albumin fluorescence at λ_ex_ = 275 nm allows for the simultaneous monitoring of tryptophanyl and tyrosyl residues, whereas the use of λ_ex_ = 295 nm almost exclusively excites the tryptophanyl residue of the macromolecules. A nearly identical course of albumin fluorescence quenching curves in GLZ-HSA (Figure 6a, in the main view) and GLZ-gHSA_FRC_ (Figure 6a, in the insert) systems, at λ_ex_ = 275 nm and λ_ex_ = 295 nm (4% difference in quenching of the intrinsic albumin fluorescence) indicates the contribution of a tryptophanyl residue or its environment and a negligible contribution of tyrosyl residues in the interaction of GLZ with both, HSA and gHSA_FRC_ in the environment of binding site. Because the HSA macromolecule contains only one tryptophanyl group (Trp-214), it can be argued that GLZ interacts with unglycated and glycated HSA mainly in subdomain IIA, but the possibility of GLZ interaction in HSA other sites cannot be excluded. This phenomenon has been confirmed by the literature data presenting experiments carried out using other than quenching fluorescence methods [39]. Because HSA contains 17 tyrosyl residues (Tyr-30, -84, -138, -140, -148, -150, -161, -263, -319, -332, -334, -341, -353, -370, -401, -411, and -497) [14], the fluorescence quenching technique is not sufficient to indicate which Tyr moieties are involved in GLZ binding.

The course of albumin fluorescence quenching curves illustrates the reduction in fluorescence intensity of unglycated and glycated albumin with the increase of gliclazide concentration in the GLZ-albumin system. For unglycated albumin, a stronger fluorescence quenching than for glycated HSA, at both excitation wavelengths λ_ex_ = 275 nm (Figure 6b, in the main view) and λ_ex_ = 295 nm (Figure 6b, in the insert) has been observed. This phenomenon indicates a greater ability of gliclazide to absorb energy from unglycated than glycated albumin. For GLZ:HSA molar ratio 20:1, the quenching of the internal HSA and gHSA_FRC_ fluorescence by GLZ equals 25.36% and 22.22%, respectively (λ_ex_ = 275 nm), while for λ_ex_ = 295 nm equals to 29.56% and 26.40%, respectively.

Literature data indicate that in the human albumin structure there are two major binding sites for compounds such as gliclazide. These sites, defined by Sudlow et al. [15] as I and II binding sites, are located in IIA subdomain (where Trp-214, Tyr-263, and His-240 are located) and IIIA (where Tyr-401, Arg-410, and Tyr-411 are located) [12]. Ryan Matsuda et al. [39], by the use of high-performance affinity chromatography (HPAC), demonstrated that gliclazide was bound to both Sudlow’s sites I and II of nonglycated and glycated HSA. Seedher and Kanojia [40] instead concluded that gliclazide can bind only in Sudlow’s sites II. They also observed that hydrophobic interactions were predominantly involved in GLZ-albumin binding. In the binding of gliclazide, albumin hydrogen bonding and electrostatic interactions took place. The results presented in this paper indicate a significant participation of Trp-214 (subdomain IIA) in GLZ-albumin complex formation, while tyrosyl residues (domains I, II, and III) are less involved. However, it should be taken into account because Trp-214 is very sensitive to structural modifications amino acid and that the distance from tryptophan (Trp-214) to the second binding site is smaller (15–17 Å) than to the first binding site (22–23 Å). It probably means that a ligand from the second binding site interacts more strongly with Trp-214 than a ligand from the site I [11]. Gliclazide probably bound to HSA and gHSA_FRC_ macromolecule near to the tryptophanyl residue affecting its amino acid environment.

**Figure 7:**
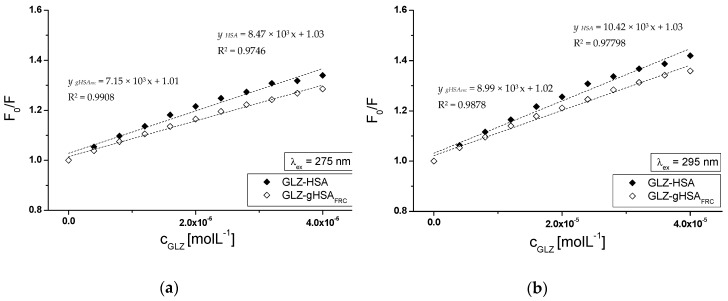
The Stern-Volmer curves for GLZ-HSA and GLZ-gHSA_FRC_. (**a**) λ_ex_ = 275 nm and (**b**) λ_ex_ = 295 nm; the error bars are smaller than the symbols.

Based on the data obtained from HSA and gHSA_FRC_ in the presence of GLZ emission fluorescence spectra, the Stern-Volmer curves ($F_{0}/F$ vs. ligand concentration) have been plotted, λ_ex_ = 275 nm (Figure 7a) and λ_ex_ = 295 nm (Figure 7b).

From $F_{0}/F=f\left(\left[C_{\mathrm{GLZ}}\right]\right)$ dependence (for GLZ-HSA and GLZ-gHSA_FRC_ systems) the Stern-Volmer $K_{\mathrm{SV}}$ and biomolecular quenching rate $k_{q}$ constants ($k_{q}=K_{\mathrm{SV}}/\tau_{0}$) have been calculated. The obtained data have been collected in Table 2.

The Stern-Volmer curves obtained for the GLZ-HSA system show a different course than the curves plotted for the GLZ-gHSA_FRC_ system, at both λ_ex_ = 275 nm (Figure 7a) and λ_ex_ = 295 nm (Figure 7b). Higher fluorescence quenching ($F_{0}/F$) in the whole range of ligand concentrations occurred for unglycated albumin compared to glycated one. The dependence of $F_{0}/F=f\left(\left[C_{\mathrm{GLZ}}\right]\right)$ showed a straight line for both, the GLZ-HSA and GLZ-gHSA_FRC_ complexes, at excitation wavelength λ_ex_ = 275 nm (Figure 7a) and λ_ex_ = 295 nm (Figure 7b). The straight-line course of Stern-Volmer dependence for the GLZ-albumin system may indicate a dynamic (collisional) or static fluorescence quenching mechanism of unglycated and glycated albumin. According to the literature data, during dynamic quenching, the ligand penetrates the environment of the macromolecule, and fluorescence quenching is caused by the collision of the quencher molecule and the fluorophore/albumin fluorophores. On the other hand, static quenching leads to a reduction in fluorescence intensity when the ligand binds to the fluorophore molecule in the ground state (unexcited) reducing the population of excitable fluorophores [42,43]. The order of the fixed fluorescence quenching rates $k_{q}$ (10^12^) determined for the GLZ-HSA and GLZ-gHSA_FRC_ system unambiguously indicates a static quenching mechanism in the gliclazide-albumin system (Table 2).

The next parameter, which was used to assess the availability of the quencher to the excited fluorophore, is the Stern-Volmer constant $K_{\mathrm{SV}}$ determined from the Stern-Volmer equation (Equation (1)). Higher values of $K_{\mathrm{SV}}$ and $k_{q}$ constants obtained for the complexes with nonglycated (HSA) than glycated (gHSA_FRC_) albumin (Table 2) indicate that gliclazide molecules locate closer to HSA than gHSA_FRC_ fluorophores. This phenomenon probably means that in a system with glycated albumin gliclazide binds at a considerable distance from fluorophores making the energy transfer difficult.

To determine the specificity of gliclazide binding to glycated and unglycated HSA, based on the Langmuir equation binding isotherms in the GLZ-HSA and GLZ-gHSA_FRC_ systems at excitation wavelengths λ_ex_ = 275 nm and λ_ex_ = 295 nm (data not shown) were plotted. The binding isotherms were determined by nonlinear regression based on the Levenberg-Marquardt algorithm. Similarly as in our previous paper [17], where the interaction between tolbutamide, losartan, and glycated albumin was studied, a nonlinear relationship $r=f\left(\left[L_{f}\right]\right)$ has been observed (data not shown). The nonlinear shape of the isotherms for GLZ-HSA and GLZ-gHSA_FRC_ complexes indicates a mixed (specific and nonspecific) nature of drugs interaction with both albumins. It probably means that nonspecific binding on HSA and gHSA_FRC_ surface or/and in the neighborhood of excited tyrosyl residues or/and formation of GLZ-HSA and GLZ-gHSA_FRC_ complexes in hydrophobic pocket of albumin takes place.

**Figure 8:**
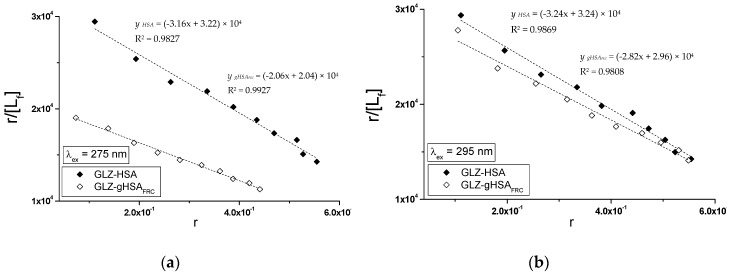
The Scatchard curves for GLZ-HSA and GLZ-gHSA_FRC_. (**a**) λ_ex_ = 275 nm and (**b**) λ_ex_ = 295 nm; the error bars are smaller than the symbols.

**Figure 9:**
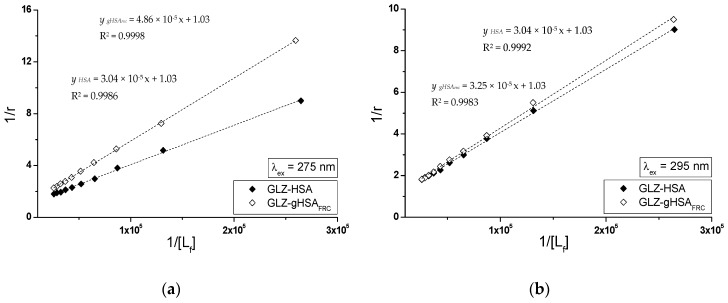
The Klotz curves for GLZ-HSA and GLZ-gHSA_FRC_. (**a**) λ_ex_ = 275 nm and (**b**) λ_ex_ = 295 nm; the error bars are smaller than the symbols.

**Figure 10:**
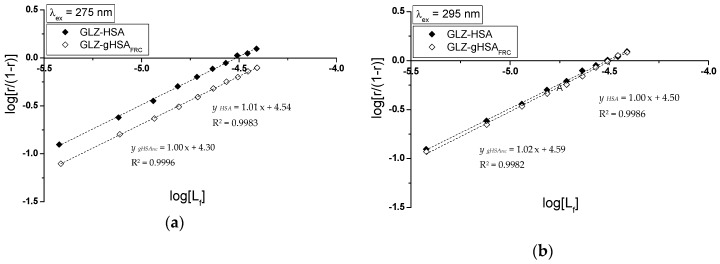
The Hill curves for GLZ-HSA and GLZ-gHSA_FRC_. (**a**) λ_ex_ = 275 nm and (**b**) λ_ex_ = 295 nm; the error bars are smaller than the symbols.

Specific binding of gliclazide to human serum albumin in GLZ-HSA and GLZ-gHSA_FRC_ complexes has been quantitatively characterized using the association constant $K_{a}$ calculated based on the Scatchard (the dependence of $r/\left[L_{f}\right]$ on r, Figure 8) and the Klotz equation (the dependence of 1/r on $1/\left[L_{f}\right]$, Figure 9). In the Scatchard equation, the concentration of the bound ligand to the protein is the independent variable (Equation (3)), while in the Klotz equation the independent variable is the reciprocal of the free ligand fraction (Equation (4)). To study the possible cooperation of ligand with albumin, the Hill equation was used (the dependence of log(r/(1−r)) on $\log\left[L_{f}\right]$, Figure 10, Equation (5)).

The changes in high affinity binding of GLZ to HSA and gHSA_FRC_ evaluated on the basis of association constants $K_{a}$ (mol^−1^∙L), the number of GLZ moles bound with 1 mole of albumin (HSA and gHSA_FRC_) the number of binding (n) and also Hill’s coefficient ($n_{H}$) obtained for GLZ-HSA and GLZ-gHSA_FRC_ systems at λ_ex_ = 275 nm and λ_ex_ = 295 nm have been calculated and summarized in Table 3.

The linear course of the Scatchard relationship (R^2^ equals to 1) for HSA and gHSA_FRC_ in the complex with GLZ at excitation wavelengths λ_ex_ = 275 nm and λ_ex_ = 295 nm (Figure 8) indicates the existence of one class of independent gliclazide binding sites in the unglycated and glycated albumin (or one binding site). Regardless of the method used (Scatchard method vs. Klotz method), glycated albumin reduces the association constant in the gliclazide-albumin system at both excitation wavelengths: λ_ex_ = 275 nm and λ_ex_ = 295 nm. Thus albumin glycation reduces the stability of GLZ-HSA complex when both the tryptophanyl and tyrosyl residues were excited. However, in both GLZ-HSA and GLZ-gHSA_FRC_ complexes a higher association value was obtained for excitation λ_ex_ = 295 nm than for λ_ex_ = 275 nm. Koyama et al. [44], using a fluorescence quenching technique, showed that the ability to bind hypoglycemic drugs to glycated albumin is lower than that to unglycated albumin at 5 × 10^−6^ mol∙L^−1^, which is consistent with the observations obtained in our study. Also, Seedher and Kanojia [40], based on the decrease in the value of association constants $K_{a}$ due to the glycation, proved that the HSA binding affinity for gliclazide, repaglinide, glimepiride, and glipizide decreases upon HSA glycation. Different conclusions were presented by Joseph et al. [45], who, when studying the in vitro effect of HSA glycation on acetohexamide binding, found an increase in the $K_{a}$ ligand association constant for glycated HSA, both at high (HAS) and low (LAS) affinity sites. The average number of gliclazide molecules bound to one unglycated and glycated molecule of albumin (n) and the Hill coefficient ($n_{H})$ interaction factors calculated for the GLZ-HSA and GLZ-gHSA_FRC_ complexes are approximately equal to 1. The value of Hill’s coefficient ≈ 1 indicates the lack of cooperativeness in the binding of gliclazide to unglycated and glycated albumin. Glycation alters the conformation of albumin due to the modification of lysine and arginine residues (Figure 1), leading to the reduction in gliclazide affinity to binding sites in the structure of the macromolecule.

#### 2.2.2. The Influence of Piracetam on the Gliclazide-Albumin Interaction

In the next part of the study, the effect of piracetam on gliclazide (glycation inhibitor) binding to transporting protein was analyzed. The emission fluorescence spectra of albumin complexed with piracetam (gHSA_FRC_-PIR) in the presence of gliclazide (GLZ), similar to gHSA_FRC_ spectrum (Figure 5), are characterized by a successive reduction in the fluorescence intensity of excited albumin fluorophores with the increase of GLZ concentration. This phenomenon indicates the absorption of energy from the excited fluorophores of albumin by gliclazide [33,34]. A hypsochromic shift of the macromolecule’s emission fluorescence in the presence of GLZ relative to the maximum emission of unbound albumin fluorescence in (gHSA_FRC_-PIR) system have been registered (data not shown). This hypsochromic shift Δλ_max_ = −4 nm (from λ_em_ = 329 nm to λ_em_ = 325 nm) and Δλ_max_ = −2 nm (from λ_em_ = 338 nm to λ_em_ = 336 nm) at the excitation wavelengths λ_ex_ = 275 nm and λ_ex_ = 295 nm, respectively, indicates an increase in the hydrophobic character of the fluorophore’s environment due to the interaction of the drug with albumin [46]. Moreover, it suggests the possibility of hydrophobic interactions between the gliclazide aromatic ring and the aromatic rings of amino acid residues located in the hydrophobic pocket (subdomains IIA, IB, IIB, and IIIA) of modified albumin in the presence of piracetam. A hypsochromic shift, greater at λ_ex_ = 275 nm than λ_ex_ = 295 nm, means that the environment of not only Trp-214 but also tyrosyl residues becomes less polar. However, a weaker shift (Δλ_max_ = 2 nm) towards “blue” due to the presence of gliclazide for gHSA_FRC_-PIR in comparison to gHSA_FRC_ (Δλ_max_ = −6 nm) (Figure 5) may indicate a decrease in the hydrophobic properties of tryptophanyl and/or tyrosyl residues environment after albumin glycation in the presence of piracetam.

**Figure 11:**
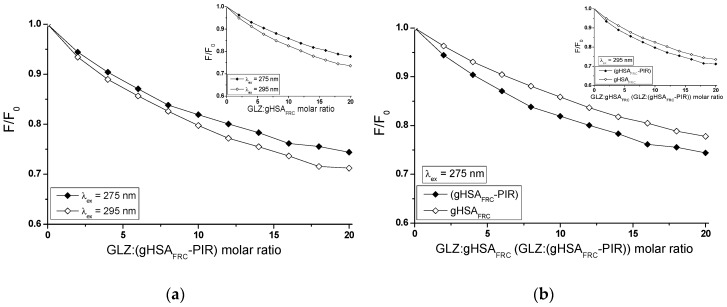
Quenching fluorescence of glycated, both in the presence (gHSA_FRC_-PIR) and absence of piracetam (gHSA_FRC_), human serum albumin complexed with GLZ (4 × 10^−6^ mol∙L^−1^–4 × 10^−5^ mol∙L^−1^). The albumins concentration was 2 × 10^−6^ mol∙L^−1^; (**a**) GLZ-(gHSA_FRC_-PIR) (in the main view) and GLZ-gHSA_FRC_ (in the insert) and (**b**) λ_ex_ = 275 nm (in the main view) and λ_ex_ = 295 nm (in the insert); the error bars are smaller than the symbols.

The glycation of human serum albumin in the presence of piracetam affects the ability of gliclazide to quench the fluorescence of macromolecule. The fluorescence quenching curves of gHSA_FRC_ and gHSA_FRC_-PIR at 2 × 10^−6^ mol∙L^−1^ concentration in the presence of gliclazide (GLZ) at 4 × 10^−6^ mol∙L^−1^–4 × 10^−5^ mol∙L^−1^ concentrations, at λ_ex_ = 275 nm and λ_ex_ = 295 nm wavelengths have been presented on Figure 11.

The course of quenching fluorescence curves illustrates the decrease in fluorescence intensity of glycated albumin, both in the absence (gHSA_FRC_) and in the presence of piracetam (gHSA_FRC_-PIR) with the increase of gliclazide concentration, at excitation wavelengths λ_ex_ = 275 nm and λ_ex_ = 295 nm (Figure 11a). For each GLZ concentration in the GLZ-gHSA_FRC_ and GLZ-(gHSA_FRC_-PIR) systems, a greater protein fluorescence quenching at λ_ex_ = 295 nm than at λ_ex_ = 275 nm has been registered. This phenomenon is probably related to the easier access of the ligand to tryptophanyl (Trp-214) than tyrosyl residues. Stronger fluorescence quenching of glycated, complexed with piracetam albumin (gHSA_FRC_-PIR) than for glycated in the absence of piracetam albumin (gHSA_FRC_), both at excitation wavelength λ_ex_ = 275 nm (Figure 11b, in the main view) and λ_ex_ = 295 nm (Figure 11b, in the insert) has been observed. This indicates a greater ability of GLZ to absorb energy from the excited macromolecule fluorophores, which is related to the reduction in the distance between the fluorophores and the ligand. Observed differences in the quenching of an internal fluorescence of albumin complexed with piracetam (gHSA_FRC_-PIR) towards albumin in the absence of piracetam (gHSA_FRC_) by GLZ are not significant and equal to 3.38% and 1.58%, both at λ_ex_ = 275 nm and λ_ex_ = 295 nm, respectively.

**Figure 12:**
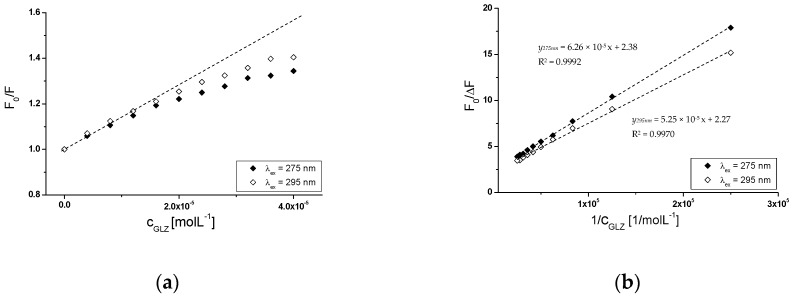
(**a**) The Stern-Volmer curves and (**b**) the Stern-Volmer curves modified by Lehrer for GLZ-(gHSA_FRC_-PIR) system; λ_ex_ = 275 nm and λ_ex_ = 295 nm; the error bars are smaller than the symbols.

Figure 12a presents Stern-Volmer curves for albumin (gHSA_FRC_-PIR) in the presence of GLZ, at excitation wavelengths λ_ex_ = 275 nm and λ_ex_ = 295 nm, respectively. An example of straight line dependence $F_{0}/F=f\left(\left[C_{\mathrm{GLZ}}\right]\right)$ has been shown in the dashed line.

Above, the GLZ:(gHSA_FRC_-PIR) molar ratio 8:1, both at λ_ex_ = 275 nm and λ_ex_ = 295 nm, the nonlinear course of the relationship described by the Stern-Volmer equation, was observed (Figure 12a). A negative deviation from the straight line dependence of $F_{0}/F=f\left(\left[C_{\mathrm{GLZ}}\right]\right)$ for the GLZ-(gHSA_FRC_-PIR) complex (λ_ex_ = 275 nm and λ_ex_ = 295 nm), indicates a mixed mechanism of quenching fluorescence. This phenomenon means that apart from dynamic (collisional) quenching, also static quenching (formation of a stable ligand-albumin complex in the ground state) has been observed. According to Eftink and Ghiron theory, a negative deviation from the straight-line course of the Stern-Volmer curve indicates that the ligand occupies first binding sites more accessible, and after saturation, more difficult to access [42,47]. The existence of dynamic and static quenching of human and bovine serum albumin fluorescence was obtained in our previous studies when the tolbutamide–albumin interaction was analyzed [17,48]. Due to the nonlinear course of Stern-Volmer dependence $F_{0}/F=f\left(\left[C_{\mathrm{GLZ}}\right]\right)$ for the GLZ-(gHSA_FRC_-PIR) system, the Lehrer modification of the original Stern–Volmer equation has been used $F_{0}/F=f\left(\left[C_{\mathrm{GLZ}}\right]\right)$ (Figure 12b). Based on the Stern-Volmer curves and equations, the Stern-Volmer constants $K_{\mathrm{SV}}$ (mol^−1^∙L) and the fraction of the initial albumin complexed with piracetam (gHSA_FRC_-PIR) fluorescence accessible to the gliclazide (quencher) ($f_{a}$), at λ_ex_ = 275 nm and λ_ex_ = 295 nm excitation wavelengths, have been calculated. Higher $K_{\mathrm{SV}}$ values $(K_{\mathrm{SV}\left(275\mathrm{nm}\right)}=$ (3.80 ± 0.06) × 10^4^ (mol^−1^∙L), $K_{\mathrm{SV}\left(295\mathrm{nm}\right)}=$ (4.32 ± 0.09) × 10^4^ (mol^−1^∙L)), calculated for GLZ-(gHSA_FRC_-PIR) system than for GLZ-gHSA_FRC_ system, ($K_{\mathrm{SV}\left(275\mathrm{nm}\right)}=$ (7.15 ± 0.22) × 10^3^ (mol^−1^∙L), $K_{\mathrm{SV}\left(295\mathrm{nm}\right)}=$ (8.99 ± 0.32) × 10^3^ (mol^−1^∙L)), have been collected in Table 2. Higher values of $K_{SV}$ constants indicate the location of gliclazide molecules closer to complexed with piracetam glycated albumin fluorophores (gHSA_FRC_-PIR) than to glycated albumin (gHSA_FRC_) fluorophores. On the basis of $f_{a}$ values analysis it was found that the presence of piracetam during HSA glycation causes the decrease in availability to tryptophanyl or/and tyrosyl resudues.

**Figure 13:**
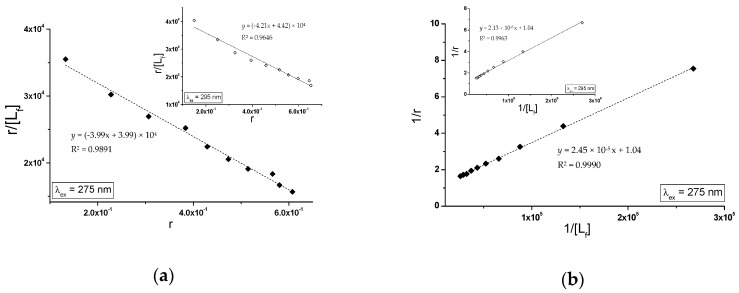
(**a**) The Scatchard curves and (**b**) the Klotz curves for GLZ-(gHSA_FRC_-PIR) system; λ_ex_ = 275 nm (in the main view) and λ_ex_ = 295 nm (in the insert); the error bars are smaller than the symbols.

In order to determine the nature of gliclazide effect on glycated albumin, in the presence of piracetam, binding isotherms in GLZ-(gHSA_FRC_-PIR) system (λ_ex_ = 275 nm and λ_ex_ = 295 nm) have been drawn. The binding isotherms determined by nonlinear regression based on the Levenberg–Marquardt algorithm showed an exponential growing course, not reaching ”plateau”, which indicates a mixed (specific and nonspecific) nature of gliclazide-albumin binding. It can therefore be assumed that GLZ binds not only to its specific binding sites in the gHSA_FRC_-PIR albumin molecule, but also nonspecifically interacts with hydrophobic surface fragments of the macromolecule. Specific binding is characterized by high affinity and low binding capacity, while nonspecific binding is characterized by low affinity and unlimited ligand binding capacity [49]. Since GLZ saturates albumin binding sites using the Scatchard (Figure 13a) and Klotz equation (Figure 13b), $K_{a}$ association constants were determined. $K_{a}$ constants determine the stability of the ligand-albumin complex, as well as the number of gliclazide molecules that form a complex with one albumin molecule in equilibrium (n). Hill’s equation was used to investigate the possible cooperation of GLZ binding to glycated albumin (gHSA_FRC_-PIR) by determining the Hill interaction coefficients ($n_{H}$).

In the quantitative analysis of gliclazide binding to glycated in the presence of piracetam albumin, the Scatchard $\left(r/\left[L_{f}\right]=f\left(r\right)\right)$ and the Klotz equations $\left(r/\left[L_{f}\right]=f\left(r\right)\right)$ were used. Hill’s equation $\left(\log\left[r/\left(1-r\right)\right]=f\left(log\left[L_{f}\right]\right)\right)$ was used to investigate the possible cooperation of GLZ binding to gHSA_FRC_-PIR.

The Scatchard model of ligand binding to the protein molecule assumes a finite number of binding sites for the ligand, then the Scatchard relationship $\left(r/\left[L_{f}\right]=f\left(r\right)\right)$ has a straight line and intersects the axis of the coordinate system (r-axis) intersection. A linear course of dependence $r/\left[L_{f}\right]=f\left(r\right)$ for the GLZ with (gHSA_FRC_-PIR) system, for both excitation wavelengths λ_ex_ = 275 nm (Figure 13a, main window) and λ_ex_ = 295 nm (Figure 13b, in the insert), indicates the existence of one class of equal, independent gliclazide binding sites in the albumin structure (or one binding site) characterized by the same association constant $K_{a}$ value. Association constants determined for the GLZ-(gHSA_FRC_-PIR) complex, both after excitation at λ_ex_ = 275 nm and λ_ex_ = 295 nm (Table 4), are higher than for the GLZ-gHSA_FRC_ complex (Table 3). The recorded mean number of gliclazide molecules bound to one molecule of glycated albumin in the presence of piracetam (n) is comparable to the n value obtained for gHSA_FRC_. The quantitative differences between the values of $K_{a}$ constants determined for the GLZ-(gHSA_FRC_-PIR) complex using the Scatchard and Klotz equations are 5.67% and 13.73%, respectively, at λ_ex_ = 275 nm and λ_ex_ = 295 nm (Table 4).

The number of binding sites (n) close to unity indicates the existence of one specific glyclazide binding site in the gHSA_FRC_-PIR and gHSA_FRC_ albumin molecule. Piracetam increases the stability of glyclazide binding by gHSA_FRC_, but at the same time does not affect the number of GLZ molecules that bind to one albumin molecule. In addition, Hill’s interaction coefficient when equal to unity ($n_{H}$ ≈ 1) indicates a lack of cooperativeness in the binding of glyclazide to albumin (gHSA_FRC_-PIR). It probably means that GLZ binding to one binding site does not affect the affinity of the ligand for other macromolecules binding sites.

## 3. Materials and Methods

### 3.1. Chemicals

Crystallized and lyophilized human serum albumin (HSA, Lot No. 8234H) with fatty acids (fraction V) was purchased from MP Biomedicals LLC (Illkirch, France). Sodium azide (NaN_3_), piracetam (PIR), and gliclazide (GLZ) were provided by Sigma-Aldrich Chemical Co. (Darmstadt, Germany). d(−)-fructose (FRC), d(+)-glucose (GLC), d(+)-galactose (GAL), Tris (hydroxymethyl)aminomethane pure p.a., and hydrochloric acid 0.1 mol∙L^−1^ (HCl) were obtained from POCH S.A. (Gliwice, Poland). All chemicals were of the highest analytical quality. The stock solution of GLZ was prepared by dissolving appropriate amounts in methanol from Merck KGaA (Darmstadt, Germany).

### 3.2. In Vitro Glycation of Human Serum Albumin

Fatted human serum albumin solution (HSA) in the presence of glucose (GLC), fructose (FRC), and galactose (GAL) was prepared in TRIS-HCl (pH 7.4) buffer solution (0.05 mol∙L^−1^) in the presence of sodium azide (NaN_3_) (0.015 mol∙L^−1^). A stock solution of piracetam (PIR) at 0.25 mol∙L^−1^ and 0.02 mol∙L^−1^ concentrations was prepared in distilled water. In order to investigate the effect of piracetam on human serum albumin glycation, HSA solutions at physiological (5 × 10^−4^ mol∙L^−1^) and 2 × 10^−6^ mol∙L^−1^ concentrations were prepared without and in the presence of piracetam (PIR) at the concentrations of 1.33 × 10^−3^ mol∙L^−1^ and 5.3 × 10^−6^ mol∙L^−1^ (the molar ratio of PIR:HSA was equal to 2.66:1). After 30 min of HSA incubation with PIR, an appropriate monosaccharide at 0.05 mol∙L^−1^ concentration was added ((HSA-PIR)-GLC, (HSA-PIR)-FRC, and (HSA-PIR)-GAL systems). All prepared solutions of proteins were passed through membrane filters with a 0.2 μm pore size, and then they were incubated in sterile closed tubes for a period of 21 days at constant temperature of 37 °C. After the incubation period, to remove the excess of unbound glucose, fructose and galactose, the solutions of gHSA_GLC_, gHSA_FRC_, gHSA_GAL_, gHSA_GLC_-PIR, gHSA_FRC_-PIR, and gHSA_GAL_-PIR, as well as control HSA solutions were dialyzed extensively against distilled water for 24 h. After dialysis, all samples were passed through 0.2 μm membrane filters and analyzed.

### 3.3. Instruments and Measurement Conditions

The fluorescence measurements of the samples were recorded at 37 °C using JASCO fluorescence spectrophotometer FP-6500 (JASCO, Easton, MD, USA) equipped with Peltier thermostat (∆T ± 0.2 °C). The instrument error for the wavelength λ was equal to ± 1.5 nm. The fluorescence spectra presented in the paper were corrected for the solvent dispersion (TRIS-HCl buffer) using the Spectra Manager program, and then analyzed using Origin version 8.5 software (Origin Northampton, MD, USA). Finally, light scattering caused by buffer was subtracted from fluorescence of samples in each spectrum using software supplied by JASCO (Spectra Manager). The results of the study were expressed as a mean ± relative standard deviation (RSD) from three independent experiments.

### 3.4. Emission Fluorescence Measurement of Human Serum Albumin and Advanced Glycation End-Products (AGEs)

Emission fluorescence spectra of AGEs glycation products in HSA-monosaccharide (2 × 10^−6^ mol∙L^−1^ and 5 × 10^−4^ mol∙L^−1^ HSA concentrations and 0.05 mol∙L^−1^ GLC, FRC, and GAL concentrations) and HSA-piracetam (PIR)-monosaccharide (2 × 10^−6^ mol∙L^−1^ HSA concentrations, 5.3 × 10^−6^ mol∙L^−1^ PIR concentrations, and 0.05 mol∙L^−1^ GLC, FRC, and GAL concentrations and 5 × 10^−4^ mol∙L^−1^ HSA concentration, 1.33 × 10^−3^ mol∙L^−1^ PIR concentration, and 0.05 mol∙L^−1^ GLC, FRC, and GAL concentrations) were registered using quartz cuvettes with optical path length 10 mm. The measuremets were made on the day of solutions preparation (HSA-GLC, HSA-FRC, HSA-GAL, (HSA-PIR)-GLC, (HSA-PIR)-FRC, and (HSA-PIR)-GAL)) and after 21 days of an incubation at 37 °C (gHSA_GLC_, gHSA_FRC_, gHSA_GAL_, gHSA_GLC_-PIR, gHSA_FRC_-PIR, and gHSA_GAL_-PIR). Fluorescence of AGEs was excited at excitation wavelength λ_ex_ = 335 nm and λ_ex_ = 370 nm (in the measurement range of 390 to 500 nm) and λ_ex_ = 485 nm (in the measurement range of 500 to 580 nm), sample scanning speed of 200 nm/min, response: 4s. The width of the excitation/emission slits was equal to 5/5 nm. However, for HSA at a concentration of 2 × 10^−6^ mol∙L^−1^ in the presence of glucose, fructose, without and in the presence of piracetam at excitation wavelength λ_ex_ = 335 nm, the width of the slits was 3/3 nm. During the measurements of AGEs fluorescence at a concentration of 2 × 10^−6^ mol∙L^−1^, the sensitivity was setted “High” (for HSA systems in the presence of glucose and galactose sensitivity was “Medium”, λ_ex_ = 370 nm). For 5 × 10^−4^ mol∙L^−1^, the sensitivity was setted to “Medium” (only for λ_ex_ = 485 nm, for HSA systems with glucose the sensitivity was “High”).

### 3.5. Emission Fluorescence Measurement of Nonmodified and Modified Human Serum Albumin in the Presence of Piracetam and Gliclazide

A stock solution of gliclazide (GLZ) at 4 × 10^−3^ mol∙L^−1^ concentration has been prepared in methanol. The content of methanol in the samples did not exceed 1% of tested protein solution total volume. The study of drug-albumin interaction has been done by albumin fluorescence quenching technique. Albumin (HSA, gHSA_FRC_, and gHSA_FRC_-PIR) fluorescence, in the absence and presence of ligand, was excited at λ_ex_ = 275 nm (exites tyrosyls and tryptophanyl residues) and λ_ex_ = 295 nm (excites tryptophanyl residue) excitation wavelengths. For the measurement standards quartz cuvettes 1 cm × 1 cm × 4 cm were used. For λ_ex_ = 275 nm and λ_ex_ = 295 nm registration range was 285 to 400 nm and 310 to 400 nm. The spectral width of the band (for monochromator of excitation and emission radiation) was equal to 5 nm, sample scanning speed of 200 nm/min, signal sensitivity “Medium”, response time: 4 s. For measurements in GLZ-HSA, GLZ-gHSA_FRC_, and GLZ-(gHSA_FRC_-PIR) systems, solutions of proteins at constant 2 × 10^−6^ mol∙L^−1^ concentration were used, without (1) and in the presence of gliclazide (GLZ) at final concentration: 4 × 10^−6^ mol∙L^−1^ (2), 8 × 10^−6^ mol∙L^−1^ (3), 12 × 10^−6^ mol∙L^−1^ (4), 16 × 10^−6^ mol∙L^−1^ (5), 20 × 10^−6^ mol∙L^−1^ (6), 24 × 10^−6^ mol∙L^−1^ (7), 28 × 10^−6^ mol∙L^−1^ (8), 32 × 10^−6^ mol∙L^−1^ (9), 36 × 10^−6^ mol∙L^−1^ (10), and 40 × 10^−6^ mol∙L^−1^ (11). Samples for fluorescence measurements were made by titration method. A suitable volume of titrant (3 μL in 10 portions) was added by the use of Hamilton syringe (10 μL) to 3 mL of unmodified and glycated HSA immediately before the fluorescence measurement. The final GLZ:HSA, GLZ:gHSA_FRC_, and GLZ:(gHSA_FRC_-PIR) molar ratio was 20:1. The degree of albumin fluorescence quenching by the drug was determined relative to the fluorescence of the nonligand albumin solutions. Absorbance of gliclazide at the used concentration was below 0.05, therefore the fluorescence spectra have not been corrected for the inner filter effect [50].

### 3.6. The Analyzis of Drug–Albumin Interaction

Using fluorescence data, the quenching curves ($F/F_{0}$ vs. GLZ:HSA, GLZ:gHSA_FRC_, or GLZ:(gHSA_FRC_-PIR), where: F and $F_{0}$ is the fluorescence intensity at the maximum wavelength of albumin in the presence and absence of a quencher, respectively) of nonglycated and glycated human serum albumin in the presence of gliclazide (GLZ) have been plotted.

The kinetics of the human serum–albumin interaction with fluorescence quenching substance was represented by the Stern-Volmer equation (Equation (1)) [51]:

(1) $$\frac{F_{0}}{F}=1+k_{q}\tau_{0}\cdot \left[C_{L}\right]=1+K_{\mathrm{SV}}\cdot \left[C_{L}\right]$$

where, $k_{q}$ is the bimolecular quenching rate constant (mol^−1^∙L∙s^−1^); $\tau_{0}$ is the average fluorescence lifetime of albumin without of quencher $\tau_{0}$ = 6.2 × 10^−9^ s [52]; $K_{\mathrm{SV}}$ is the Stern-Volmer constant (mol^−1^∙L); [$C_{L}$] is the ligand concentration (mol∙L^−1^); $\left[L\right]=\left[L_{b}\right]+[L_{f}]$, $\left[L_{b}\right]$, and $\left[L_{f}\right]$ are the bound and free (unbound) drug concentrations (mol∙L^−1^).

A straight line course of Stern-Volmer dependence (1) takes place during both the dynamic, and the static mechanism of fluorescence quenching in the ligand–protein system. This means that the fluorophores of fluorescing molecule are equally available for the quencher [47]. Whereas, when the Stern-Volmer dependence is not a straight line, a positive deviation from straightness indicates the presence of a mixed mechanism (static and dynamic) of fluorescence quenching however the negative deviation may be the reason of two populations of fluorophores that differ in availability for the quencher [43]. Nonlinear quenching of fluorescence can be described by Lehrer’s modified Stern-Volmer equation (Equation (2)). Based on this, not only Stern-Volmer $K_{\mathrm{SV}}$ constants as well as the fractional maximum protein fluorescence accessible for the quencher were determined [53].

(2) $$\frac{F_{0}}{\Delta F}=\frac{1}{\left[C_{L}\right]}\cdot \frac{1}{f_{a}}\cdot \frac{1}{K_{\mathrm{SV}}}+\frac{1}{f_{a}}$$

where, ΔF is the difference between $F_{0}$ and F and $f_{a}$ is the fractional maximum protein fluorescence accessible for the quencher.

Isotherms of drug binding to HSA, gHSA_FRC_ and gHSA_FRC_-PIR have been obtained based on the graph of the function $r=f\left(\left[L_{f}\right]\right)$, where, $r=\frac{\left[L_{b}\right]}{\left[HSA\right]}$ is the number of ligands moles bound per mole of protein molecule; $\left[L_{b}\right]=\frac{\Delta F}{\Delta F_{\max}}\cdot HSA_{\mathrm{total}}$, ΔF is the difference between $F_{0}$ and F, $\Delta F_{\max}$ (maximal fluorescence change with complete saturation) is evaluated from the linear part of the $\frac{1}{\Delta F}$ vs. $\frac{1}{\left[L\right]}$; [HSA] is serum albumin concentration (mol∙L^−1^) [49].

In order to obtain association constants values for drug-albumin $K_{a}$ complex and the number of binding sites for the independent class of drug binding sites in the albumin molecule (n) the Scatchard (Equation (3)) [54] and Klotz (Equation (4)) equations were used [55]:

(3) $$\frac{r}{\left[L_{f}\right]}=n\cdot K_{a}-K_{a}\cdot r$$

(4) $$\frac{1}{r}=\frac{1}{n}+\frac{1}{n\cdot K_{a}\cdot [L_{f}]}$$

A graphical illustration of the relationship described by the formula (3) is the Scatchard curve. The tangent to the Scatchard curve at the intersection with the X-axis represents the average number of moles of ligand bound to one mole of albumin in the analyzed binding site. A linear Scatchard curve indicates the existence of one, independent class of binding sites in the albumin molecule. The Scatchard curve can also have a nonlinear curve. The course of the curve similar to hyperbole indicates the nonspecific nature of ligand binding, negative cooperativeness or the existence of many classes of binding sites. A “conical” curve indicates positive cooperativity or instability of the ligand [56].

Hill’s coefficient was determined on the basis of Hill’s method (Equation (5)) [57]:

(5) $$log\left(\frac{r}{1-r}\right)=n_{H}\cdot log[L_{f}]+logK_{a}$$

where, $n_{H}$ is the Hill’s coefficient. For $n_{H}$ = 1 the binding of the ligand to the macromolecule is completely noncooperative. For $n_{H}$ > 1 there is a positive cooperative relationship: binding the ligand in one place increases the affinity of the ligand to the rest of protein molecule binding sites. $n_{H}$ < 1 means a reduction in the affinity of the ligands to the next binding site.

## 4. Conclusions

The primary objective and novelty of this study was to estimate the inhibition properites of piracetam (PIR) and its impact on the gliclazide (GLZ)–glycated albumin interaction. Based on the conducted in vitro data we concluded that piracetam (PIR) used in eldery simultaneously with gliclazide (GLZ) inhibits the formation of Advanced Glycation Ends Products (AGEs) and increases the binding strength of GLZ to glycated albumin that weakens its therapeutic effect. Although the studies are preliminary and cannot be directly used in clinical practice, the results highlight the novelty and validity of the studies and suggest using other research methods as a continuation of this work.

## Figures and Tables

**Table 1:** The quotient of AGEs fluorescence intensity in gHSA_GLC_, gHSA_FRC_, gHSA_GAL_, gHSA_GLC_-PIR, gHSA_FRC_-PIR, gHSA_GAL_-PIR, [HSA] 2 × 10^−6^ mol∙L^−1^ and 5 × 10^−4^ mol∙L^−1^, λ_ex_ = 335 nm, λ_ex_ = 370 nm, and λ_ex_ = 485 nm.

[C_albumin_]	$\frac{F_{gHSA_{monosaccharide}}}{F_{gHSA_{monosaccharide}-PIR}}$	λ_ex_ = 335 nm	λ_ex_ = 370 nm	λ_ex_ = 485 nm
2 × 10^−6^ mol∙L^−1^	$\frac{{\mathrm{gHSA}}_{\mathrm{GLC}}}{{\mathrm{gHSA}}_{\mathrm{GLC}}-\mathrm{PIR}}$	1.81	2.28	1.24
	$\frac{{\mathrm{gHSA}}_{\mathrm{FRC}}}{{\mathrm{gHSA}}_{\mathrm{FRC}}-\mathrm{PIR}}$	2.66	3.11	3.42
	$\frac{{\mathrm{gHSA}}_{\mathrm{GAL}}}{{\mathrm{gHSA}}_{\mathrm{GAL}}-\mathrm{PIR}}$	45.70	30.28	29.56
5 × 10^−4^ mol∙L^−1^	$\frac{{\mathrm{gHSA}}_{\mathrm{GLC}}}{{\mathrm{gHSA}}_{\mathrm{GLC}}-\mathrm{PIR}}$	1.17	1.11	1.10
	$\frac{{\mathrm{gHSA}}_{\mathrm{FRC}}}{{\mathrm{gHSA}}_{\mathrm{FRC}}-\mathrm{PIR}}$	1.22	1.22	1.12
	$\frac{{\mathrm{gHSA}}_{\mathrm{GAL}}}{{\mathrm{gHSA}}_{\mathrm{GAL}}-\mathrm{PIR}}$	1.38	1.48	1.13

**Table 2:** Stern-Volmer constants $K_{SV}$ (mol^−1^∙L) and biomolecular quenching rate constants $k_{q}$ (mol^−1^∙L∙s^−1^) calculated for GLZ-HSA and GLZ-gHSA_FRC_ systems; λ_ex_ = 275 nm and λ_ex_ = 295 nm.

**λ_ex_ = 275 nm**	$K_{SV}$ ± RSD *^)^ × 10^3^ (mol^−1^∙L)	^a^$k_{q}$ ± RSD *^)^ × 10^12^(mol^−1^∙L∙s^−1^)
GLZ-HSA	8.47 ± 0.43	1.37 ± 0.07
GLZ-gHSA_FRC_	7.15 ± 0.22	1.15 ± 0.04
**λ_ex_ = 295 nm**	$K_{SV}$ ± RSD *^)^ × 10^3^ (mol^−1^∙L)	^a^$k_{q}$ ± RSD *^)^ × 10^12^ (mol^−1^∙L∙s^−1^)
GLZ-HSA	10.42 ± 0.49	1.68 ± 0.08
GLZ-gHSA_FRC_	8.99 ± 0.32	1.45 ± 0.05

*^)^ relative standard deviation; ^a^ calculated using: $k_{q}=\frac{K_{SV}}{\tau_{0}}$, where: $\tau_{0}$ = 6.2 × 10^−9^ s [41]—the average fluorescence lifetime of albumin without quencher.

**Table 3:** Association constants $K_{a}$ (mol^−1^∙L), mean number of GLZ moles bound with one mole of HSA and gHSA_FRC_ (n), and the Hill’s coefficient ($n_{H}$) in GLZ-HSA and GLZ-gHSA_FRC_ systems; λ_ex_ = 275 nm and λ_ex_ = 295 nm.

	Scatchard Method		Klotz Method		Hill Method
λ_ex_ = 275 nm	$K_{a}$ ± RSD *^)^ × 10^4^ (mol^−1^∙L)	n ± RSD *^)^	$K_{a}$ ± RSD *^)^ × 10^4^ (mol^−1^∙L)	n ± RSD *^)^	$n_{H}$ ± RSD *^)^
GLZ-HSA	3.16 ± 0.14	1.02 ± 0.06	3.38 ± 0.09	0.97 ± 0.04	1.01 ± 0.01
GLZ-gHSA_FRC_	2.06 ± 0.06	0.99 ± 0.04	2.11 ± 0.04	0.97 ± 0.02	1.00 ± 0.01
λ_ex_ = 295 nm	$K_{a}$ ± RSD *^)^ × 10^4^ (mol^−1^∙L)	n ± RSD *^)^	$K_{a}$ ± RSD *^)^ × 10^4^ (mol^−1^∙L)	n ± RSD *^)^	$n_{H}$ ± RSD *^)^
GLZ-HSA	3.24 ± 0.12	1.00 ± 0.05	3.38 ± 0.06	0.97 ± 0.03	1.00 ± 0.01
GLZ-gHSA_FRC_	2.82 ± 0.13	1.05 ± 0.07	3.17 ± 0.10	0.97 ± 0.04	1.02 ± 0.02

*^)^ relative standard deviation.

**Table 4:** Association constants $K_{a}$ (mol^−1^∙L), mean number of GLZ molecule bound with one molecule of gHSA_FRC_-PIR (n), and the Hill’s coefficient $\left(n_{H}\right)$ in the gHSA_FRC_-PIR system; λ_ex_ = 275 nm and λ_ex_ = 295 nm.

	Scatchard Method		Klotz Method		Hill Method
(gHSA_FRC_-PIR)	$K_{a}$ ± RSD *^)^ × 10^4^ (mol^−1^∙L)	n ± RSD *^)^	$K_{a}$ ± RSD *^)^ × 10^4^ (mol^−1^∙L)	n ± RSD *^)^	$n_{H}$ ± RSD *^)^
λ_ex_ = 275 nm	3.99 ± 0.14	1.00 ± 0.05	4.23 ± 0.07	0.97 ± 0.02	1.00 ± 0.01
λ_ex_ = 295 nm	4.21 ± 0.27	1.05 ± 0.10	4.88 ± 0.12	0.96 ± 0.04	1.04 ± 0.03

*^)^ relative standard deviation.
